# Supplementary material for: Efficacy and Safety of Leflunomide for Refractory COVID-19: A Pilot Study
Source: Front Pharmacol. 2021 Jul 2;12:581833. doi: 10.3389/fphar.2021.581833 (PMC8284962; doi:10.3389/fphar.2021.581833)
Supplement: Supplementary file 1 [file DataSheet1.docx]

**Supplementary Material**

**Supplementary figure legends**

Figure S1. The Kaplan-Meier Curve of Time to SARS-CoV-2 Clearance in Sensitive Analysis.

Figure S2. The Kaplan-Meier Curve of Time to Discharge from Hospital in Sensitive Analysis.

Figure S3. Time Course of Statuses of SARS-CoV-2 Test and End Points in Sensitive Analysis.


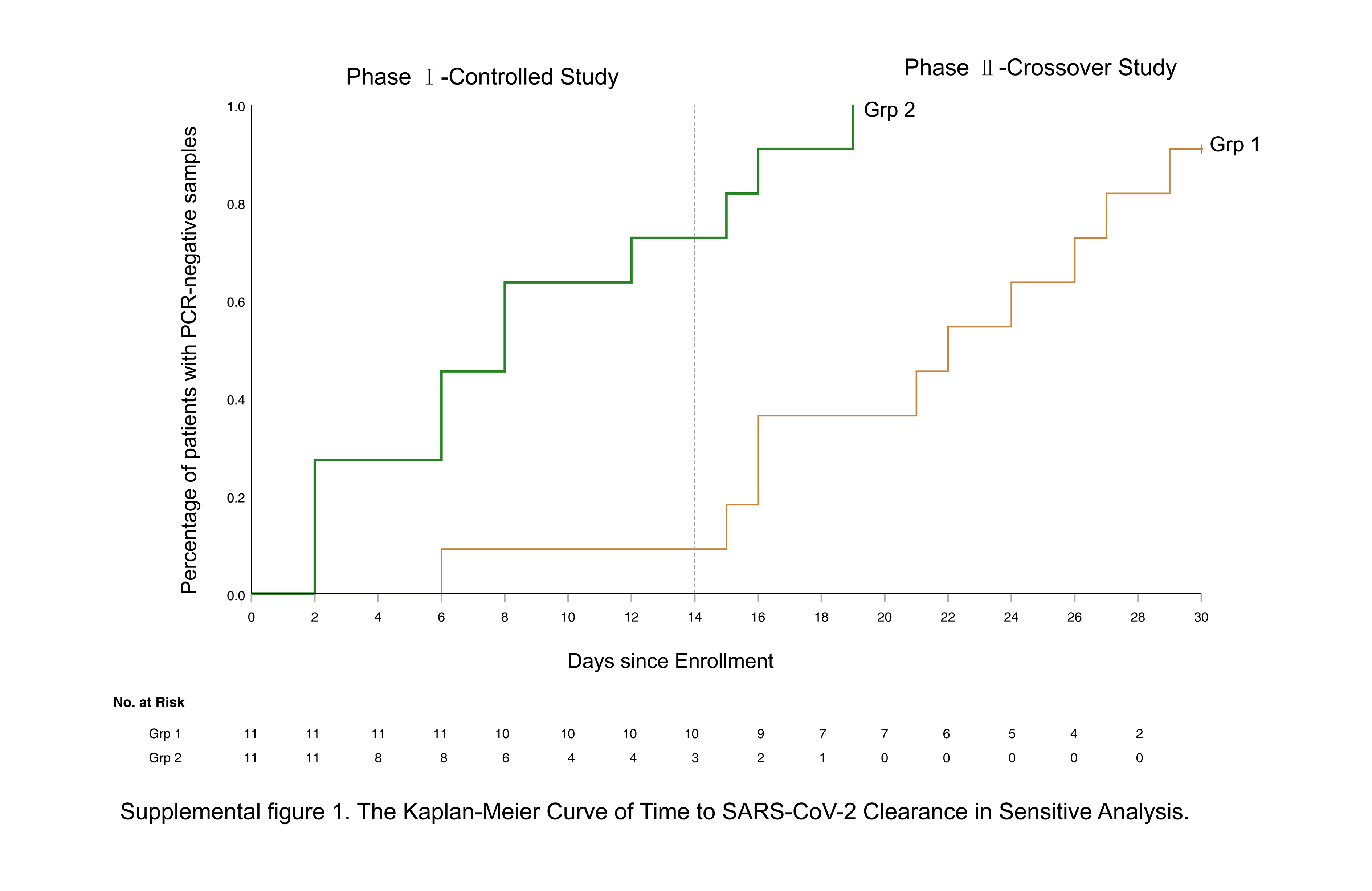


**Figure S1. The Kaplan-Meier Curve of Time to SARS-CoV-2 Clearance in Sensitive Analysis.**


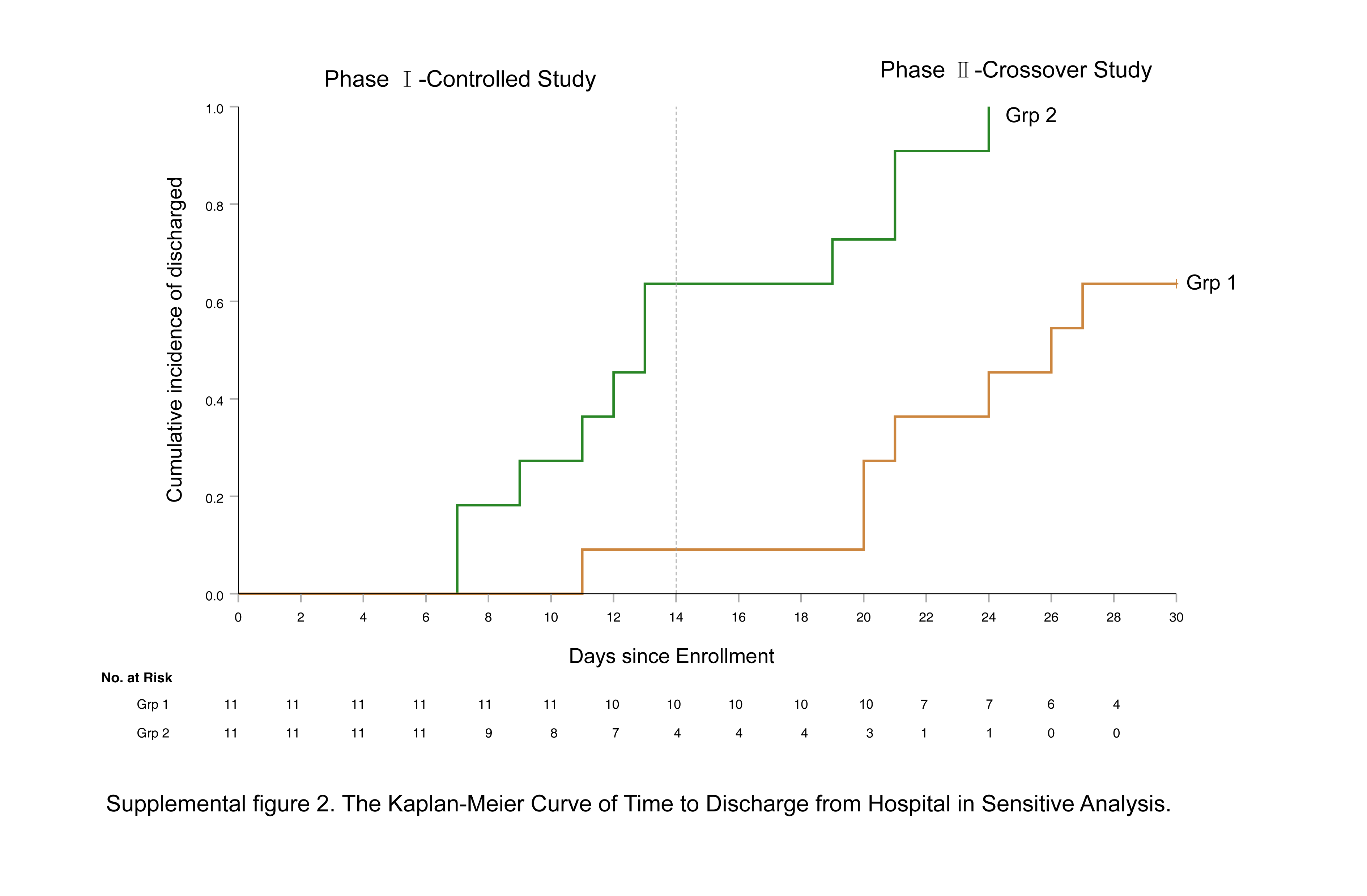


**Figure S2. The Kaplan-Meier Curve of Time to Discharge from Hospital in Sensitive Analysis.**


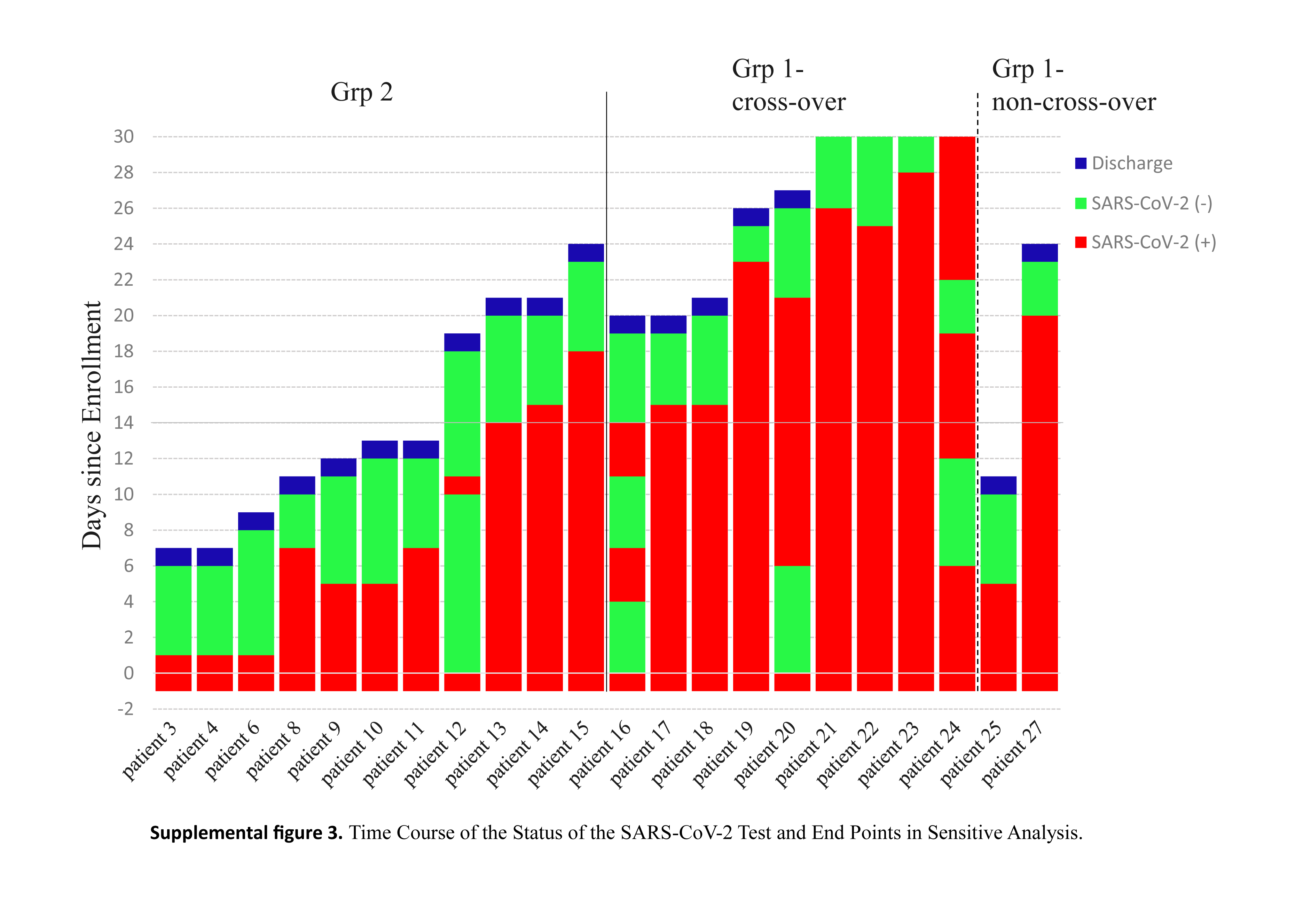


**Figure S3. Time Course of the Status of the SARS-CoV-2 Test and End Points in Sensitive Analysis.**

**Table S1.** **Patient Pharmacotherapeutic Characteristics***

| Medication | Group 2 (Leflunomide)  (N = 15) | Group 1 (SOC)  (N = 12) | P  Value | Total  (N = 27) |
| --- | --- | --- | --- | --- |
| Antibiotics |  |  |  |  |
| Azithromycin | 4 (26.7) | 4 (33.3) | 1.00 | 8 (29.6) |
| Others | 11 (73.3) | 11 (91.7) | 0.34 | 22 (81.5) |
| Antiviral drugs |  |  |  |  |
| Interferon-α | 10 (66.7) | 10 (83.3) | 0.41 | 20 (74.1) |
| Arbidol | 14(93.3) | 11 (91.7) | 1.00 | 25 (92.6) |
| Oseltamivir | 4 (26.7) | 9 (75.0) | 0.02 | 13 (48.1) |
| Ribavirin | 6 (40) | 6 (50.0) | 0.71 | 12 (44.4) |
| Lopinavir/ritonavir | 0 | 1 (8.3) | 0.44 | 1 (3.7) |
| Ganciclovir | 0 | 1 (8.3) | 0.44 | 1 (3.7) |
| Hydroxychloroquine | 11 (73.3) | 11 (91.7) | 0.34 | 22 (81.5) |
| Glucocorticoids | 7 (46.7) | 4 (33.3) | 0.7 | 11 (40.7) |
| Thymalfasin | 14 (93.3) | 9 (75.0) | 0.29 | 23 (85.2) |
| COVID-19 convalescent plasma | 0 | 1 (8.3) | 0.44 | 1 (3.7) |
| Intravenous immunoglobulins | 3 (20) | 2 (16.7) | 1.00 | 5 (18.5) |
| Traditional Chinese herbal medicine |  |  |  |  |
| Lianhua Qingwen capsule | 12 (80) | 11 (91.7) | 0.61 | 23 (85.2) |

*****Data are shown in n (%).

**Table S2.** **Treatment Emergent Adverse Events (TEAEs) in the Study Population***

| Event | Group 2(Leflunomide)  (N = 15) | Group 1 (SOC)  (N = 12) |
| --- | --- | --- |
| Any adverse events | 6 (40) | 3 (25) |
| Hyperlipidemia | 3 (20) | 2 (16.7) |
| Leukopenia | 3 (20) | 0 |
| Neutropenia | 2 (13.3) | 0 |
| ALT elevations | 1 (6.7) | 0 |
| Lymphopenia | 1 (6.7) | 0 |
| Thrombocytopenia | 1 (6.7) | 0 |
| Hypoalbuminemia | 0 | 1 (8.3) |

*****Data are shown in n (%).
